# Reduced endocytosis and altered lysosome function in cisplatin-resistant cell lines

**DOI:** 10.1038/sj.bjc.6600861

**Published:** 2003-04-15

**Authors:** S S Chauhan, X J Liang, A W Su, A Pai-Panandiker, D W Shen, J A Hanover, M M Gottesman

**Affiliations:** 1 Laboratory of Cell Biology, Center for Cancer Research, National Cancer Institute, National Institutes of Health, 37 Convent Dr, Room 1A09, Bethesda, MD 20842-4254, USA; 2 Laboratory of Cell Biochemistry and Biology, National Institute of Diabetes and Digestive and Kidney Diseases, National Institutes of Health, 8 Center Dr., Room 402, Bethesda, MD 20892-0850, USA

**Keywords:** cisplatin-resistance, EGF binding, fluid-phase/receptor-mediated endocytosis, endosomal/lysosomal acidification, *Pseudomonas* exotoxin

## Abstract

We isolated human KB adenocarcinoma cisplatin-resistant (CP-r) cell lines with multidrug-resistance phenotypes because of reduced accumulation of cisplatin and other cytotoxic compounds such as methotrexate and heavy metals. The uptake of horseradish peroxidase (HRPO) and Texas Red dextran was decreased several-fold in KB-CP-r cells, indicating a general defect in fluid-phase endocytosis. In contrast, although EGF receptors were decreased in amount, the kinetics of EGF uptake, a marker of receptor-mediated endocytosis, was similar in sensitive and resistant cells. However, 40–60% of the ^125^I-EGF released into the medium after uptake into lysosomes of KB-CP-r cells was TCA precipitable as compared to only 10% released by sensitive cells. These results indicate inefficient degradation of internalised ^125^I-EGF in the lysosomes of KB-CP-r cells, consistent with slower processing of cathepsin L, a lysosomal cysteine protease. Treatment of KB cells by bafilomycin A_1_, a known inhibitor of the vacuolar proton pump, mimicked the phenotype seen in KB-CP-r cells with reduced uptake of HRPO, ^125^I-EGF, ^14^C-carboplatin, and release of TCA precipitable ^125^I-EGF. KB-CP-r cells also had less acidic lysosomes. KB-CP-r cells were crossresistant to *Pseudomona*s exotoxin, and *Pseudomonas* exotoxin-resistant KB cells were crossresistant to cisplatin. Since cells with endosomal acidification defects are known to be resistant to *Pseudomonas* exotoxin and blocking of endosomal acidification mimics the CP-r phenotype, we conclude that defective endosomal acidification may contribute to acquired cisplatin resistance.

Cisplatin (CP) is a component of standard treatment regimens for testicular, ovarian, bladder, cervical, head and neck and small-cell and nonsmall-cell lung cancers (Ozols and Williams, 1989; Perez, 1998). Adducts of DNA with CP induce apoptosis leading to cell death (Minn *et al*, 1995; Simonian *et al*, 1997). Intrinsic or acquired resistance of tumour cells to CP undermines its clinical effectiveness (Niedner *et al*, 2001). Mechanisms of resistance include decreased drug accumulation (Shen *et al*, 2000), changes in DNA repair proficiency (Fink *et al*, 1998; Zhen *et al*, 1992; Chu, 1994; Lai *et al*, 1995; Johnson *et al*, 1996), metallothionein (MT II) (Kelly *et al*, 1988; Kondo *et al*, 1995), glutathione-related enzymes (Moscow and Cowan, 1988; Godwin *et al*, 1992; Zaman *et al*, 1995), stress response proteins (Shen *et al*, 1995; Hettinga *et al*, 1997), proto-oncogenes or apoptosis-related genes, and cancer susceptibility genes (Fanidi *et al*, 1993; Lowe *et al*, 1993; DeFeudis *et al*, 1996; Husain *et al*, 1998; Moorehead and Singh, 2000). Protein kinases (PKs) like PKA and PKC have also been associated with CP-resistance (CP-r) and use of their specific inhibitors has been demonstrated to increase CP cytotoxicity in resistant tumour cells (Gosland *et al*, 1996). Recently, our laboratory (Shen *et al*, 2000) reported decreased energy-dependent uptake of ^14^C-carboplatin by CP-r cells. Copper transporters have recently been described to be involved in cisplatin uptake in yeast and mouse (Ishida *et al*, 2002), and in human cell lines (Katano *et al*, 2002). However, we have been unable to demonstrate any alternation in expression of the copper transporter CTR1 in our CP-r cell lines, and expression of CTR1 in our cells does not change cisplatin accumulation (data not shown). It was of interest to evaluate various uptake pathways in CP-r cells.

Results of the present study demonstrate the reduced uptake of fluid-phase endocytotic markers by CP-r cells. We also report here the incomplete degradation of internalised EGF and slow processing of lysosomal cysteine protease cathepsin L in CP-r cells. Treatment of normal cells with the vacuolar proton pump inhibitor bafilomycin A_1_ mimics the CP-r phenotype in wild-type cells, suggesting that an endosomal/lysosomal acidification defect or another similar defect in the endocytic pathway may be plausible mechanisms contributing to CP-r.

## MATERIALS AND METHODS

### Cell lines and cell culture

The previously described human epidermoid carcinoma cell line KB-3-1 (IC_50_ 0.1 *μ*g ml^−1^ cisplatin) and its CP-r derivative KB-CP20 (selected for resistance to 20 *μ*g ml^−1^ cisplatin in many steps) were used in the present study. These cells were grown as monolayer cultures in Dulbecco's modified eagle's medium (Quality Biological, Gaithersburg, MD, USA) containing 4.5 g l^−1^ glucose (Gibco-BRL, Grand Island, NY, USA), glutamine, penicillin, streptomycin and 10% foetal bovine serum at 37°C in a CO_2_ incubator. The KB-CP20 cells were maintained in the above culture medium containing CP (20 *μ*g ml^−1^) as described earlier (Shen *et al*, 1995). Cisplatin was removed from the culture medium 3 days prior to conducting experiments. KB-CP20-EGFR-cl-6 cells were cloned from transfectants of KB-CP20 cells with the pcDNA/EGFR expression vector, and used for studies on EGF binding and uptake. *Pseudomonas* exotoxin-resistant cell lines, ET-12, ET-22 and ET-28 (ET: EGF-toxin-resistant cells), were established and described in detail by Amano *et al* (1988). These ET cell lines were up to more than 10 000-fold resistant to PE-EGF conjugate in comparison with their parent wild-type KB-3-1 cells as determined by a colony-forming assay.

### Uptake assays of horse radish peroxidase (HRPO) and Texas Red dextran-10

For the HRPO assay, KB-3-1 or KB-CP20 cells were seeded in each well of a six-well plate 18 – 24 h before the assay. The next day, the cells were washed three times with serum-free medium and incubated at 37°C with medium containing 2 mg ml^−1^ HRPO type VI. After various intervals of time, the uptake medium was removed and the cells were washed several times with ice-cold phosphate-buffered saline (PBS), and lysed in PBS containing 0.2% TritonX-100. The cell lysate was centrifuged (12 000 **g**) in a microcentrifuge at 4°C and HRPO was assayed in the supernatant fraction by the method of West *et al* (1989). For uptake of Texas Red dextran-10 (Molecular Probes, Eugene, OR, USA), both KB-3-1 and KB-CP20 cells were incubated with this fluorescence-labelled marker at 37°C for 2 h, and then monitored under a laser scanning confocal microscope (Bio-Rad, Hercules, CA, USA) at a × 600 magnification. A time course for influx of Texas Red dextran-10 was performed by incubation of cells with 3 mg ml^−1^ of this fluorescence marker at a desired period of time, and then analysed by a FACSort flow cytometer (Becton Dickinson, Franklin Lakes, NJ, USA) equipped with Cell Quest software.

### ^14^C-carboplatin uptake assay and metabolic labelling

The uptake of ^14^C-carboplatin was measured essentially by the method described earlier (Shen *et al*, 2000). Briefly, 2 × 10^6^ cells were plated in each well of a six-well Petri dish. The next day, the cells were washed with prewarmed DMEM and incubated with ^14^C-carboplatin in DMEM (2 mCi ml^−1^). After 1 h, medium was removed, cells were washed 3 × with ice-cold PBS and harvested by trypsinisation. ^14^C-carboplatin taken up by cells was quantitated using a Beckman Liquid Scintillation Counter (LS 2800, Fullerton, CA, USA) after solubilising them in Formula 989 (Dupont, NEN, Boston, MA, USA). For metabolic labelling, 2 × 10^6^ cells were plated in each well of a six-well Petri dish. The next day, the cells were washed twice with methionine-free, cysteine-free medium containing 2 mM glutamine and preincubated in the same medium for 1 h before labelling in the presence of ^35^S-translabel (200 *μ*Ci ml^−1^). After 30 min, the cells were washed three times with ice-cold PBS and either lysed in SDS buffer A (50 mM Tris-HCl pH 7.4, 150 mM KCl, 0.5% NP-40 and 0.05% SDS) immediately or fed with regular serum-free medium. After 30, 60, 120 and 240 min, the culture medium was collected. The cells were washed three times with ice-cold PBS and lysed. The cell lysate of the 30 min pulse period containing 1 × 10^6^ TCA precipitable counts, and an equal volume of lysate were used at the other chase periods. The lysates were immunoprecipitated with rabbit polyclonal antibody to the lysosomal cysteine protease cathepsin L as described previously (Chauhan *et al*, 1998). Similarly, secreted cathepsin L in a volume of culture medium proportional to the amount of lysate from different chase periods was also immunoprecipitated. The immunoprecipitates were resolved on SDS – PAGE and subjected to autoradiography as described earlier (Chauhan *et al*, 1998). The 42 kDa unprocessed form of human cathepsin L or its 34 and 26 kDa processed forms were quantitated by a phosphorImager 425 (Molecular Dynamics, Sunnyvale, CA, USA).

### Surface binding of ^125^I-EGF

KB-3-1 and KB-CP20 cells were plated in triplicate in 24-well tissue culture dishes at a cell density of 2 × 10^5^ cells well^−1^ in 0.5 ml containing 10% FBS. The following day, the cells were fed with fresh DMEM and incubated for 1 h at 37°C. Then the cells were chilled on ice for 30 min and ^125^I-EGF (8 nM, specific activity 75 Ci mmol^−1^) was added to each well. The incubation was continued on ice for an additional 2 h. Then the medium was aspirated off, cells were washed three times with ice-cold PBS and solubilised in 0.5 ml of 1 N NaOH, and ^125^I-EGF bound to the cell surface was determined by using a mini-gamma counter (LKB, Gaithersburg, MD, USA). To determine the nonspecific binding of radiolabelled EGF, cells were incubated with ^125^I-EGF in the presence of a 10 M excess of unlabelled EGF and processed similarly. This value was subtracted from the specific binding. The nonspecific binding was never observed to be more than 5% of the specific binding.

### Internalisation, degradation and release of ^125^I-EGF

Measurement of internalisation and degradation of radiolabelled EGF by CP-r and cisplatin-sensitive (CP-s) cells was performed as described earlier (Das *et al*, 1989). ^125^I-EGF was allowed to bind to surface receptors at 4°C for 2 h as described above. While still on ice, the cells were washed with ice-cold PBS three times and fed with 1.0 ml complete media followed by incubation at 37°C in a CO_2_ incubator for 0, 5, 15, 30, 60 and 120 min. At the end of each time period, the medium was saved and total and TCA precipitable radioactivity was determined. The cells were washed with ice-cold PBS and incubated on ice for 6 min with 1.0 ml of mild acid (0.2 M acetic acid – 0.5 M NaCl, pH 2.5). The acidic medium was carefully removed and counted (acid-dissociated radioactivity). The remaining cell-associated radioactivity was solubilised at 60°C with 1.0 ml of 1 N NaOH and counted in a gamma counter (nondissociated radioactivity).

### Drug-sensitivity assay

Dose – response curves were determined by seeding 5 × 10^4^ cells in 1 ml of medium in each well of a 24-well plate. Drugs at desired concentrations were introduced into the wells at the time of cell seeding. After 37°C incubation for 3 days, cells were counted by a Coulter Counter. An IC_50_ was measured as the concentration of drug reducing the growth of cells to 50% of that in control (drug-free) medium. A relative resistance factor value for each drug was calculated by dividing the IC_50_ value of each cell line by that of the wild-type KB-3-1 cells. The values are means of triplicate determinations.

### Measurement of endosomal and lysosomal pH

Cells (200 ml) at a density of 1 × 10^6^ cells ml^−1^ were placed onto an 18 × 18 mm coverslip and allowed to grow for 3 days. KB-3-1 and KB-CP20 cells were incubated with 1.5 mM LysoSensor DND-189 (Molecular Probes, Eugene, OR, USA) in DMEM medium for 45 min, and then replaced with fresh medium. Acidified compartments were visualised by laser confocal microscopy. pH determination by LysoSensor-binding fluorescence studies was performed according to the manufacturer's instructions. For analysis of lysosomal distribution, cells were incubated with 100 nM LysoTracker Red DND-99 (Molecular Probes, Eugene, OR, USA) for 30 min, then monitored under a laser scanning confocal microscope as described above. For direct measurement of endosomal pH, cellubrevin pHluorins were applied as described by Miesenbock *et al* (1998).

## RESULTS

### Measurement of fluid-phase endocytosis

**Figure 1:**
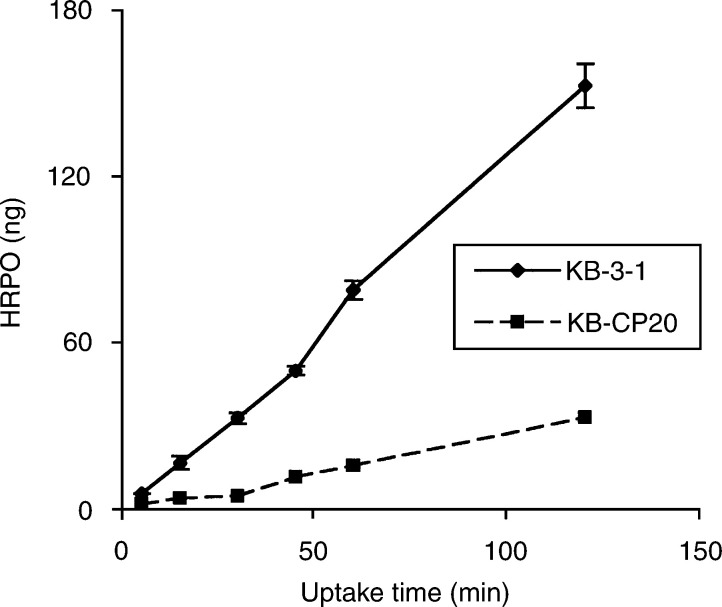
Kinetics of HRPO uptake by KB-3-1 and KB-CP20 cells. Cells were incubated with 2 mg ml^−1^ HRP at 37°C. After various time intervals, the cells were washed several times, lysed in PBS containing 0.2% Triton X-100, and HRP in the cell lysate was assayed. Values expressed are mean ±s.d. from three independent experiments.

To determine nonreceptor-mediated fluid-phase endocytosis, we measured uptake of HRPO at different intervals of time ranging from 5 to 120 min to assess fluid-phase endocytosis in CS-s (KB-3-1) and Cs-r (KB-CP20) cells, and the results are given in Figure 1

. We observed a five-fold decrease in the uptake of HRPO by KB-CP20 cells as compared to their sensitive counterparts. This difference in HRPO uptake was consistently seen at all intervals of time ranging from 5 to 120 min.

**Figure 2:**
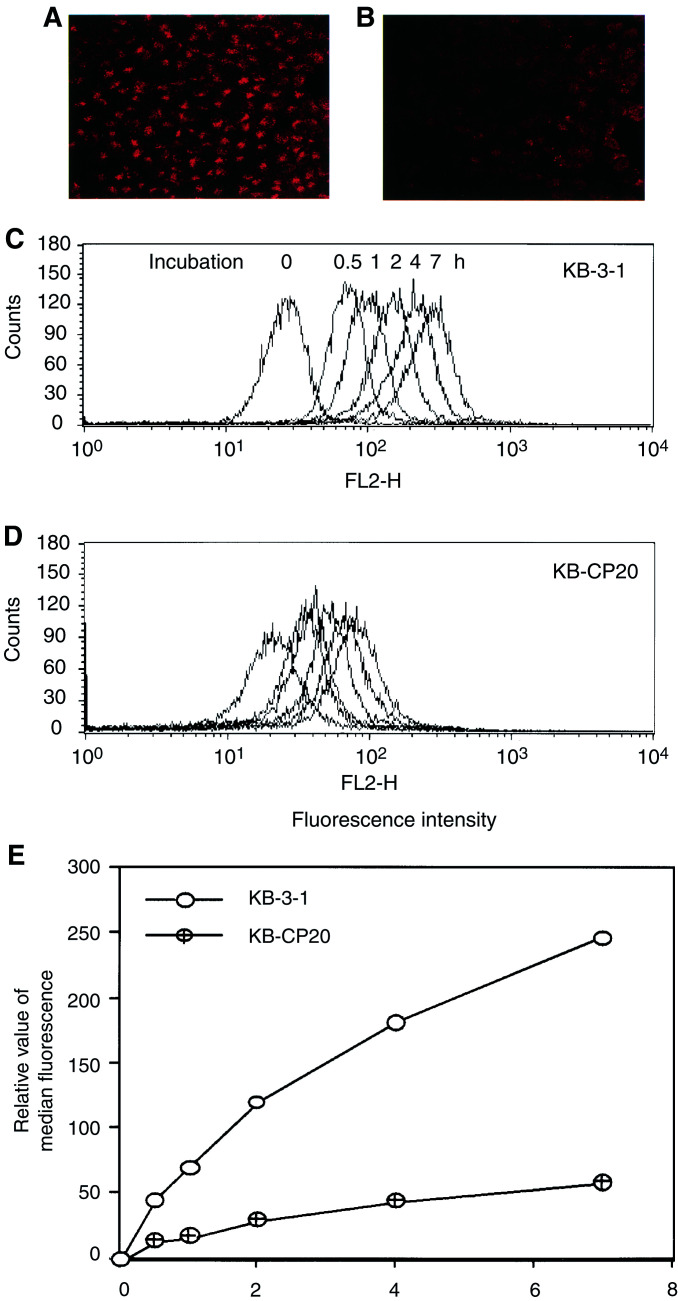
Reduced accumulation of Texas Red dextran-10, a fluid-phase marker, in CP-r cells. (**A**, **B**) Cells were incubated with 10 mg ml^−1^ of Texas Red dextran-10 at 37°C for 2 h, then monitored under a laser scanning confocal microscope (Bio-Rad, Molecular Probes, Eugene, OR, USA) at a x600 magnification. (**C**, **D**) A time course of Texas Red dextran-10 accumulation was performed by FACS analysis. Cells were incubated with 3 mg ml^−1^ of Texas Red dextran-10 at 37°C for up to 7 h. (**E**) A semiquantitative analysis based on the data of Figure 2C and D.

Accumulation of Texas Red dextran-10, another fluid-phase endocytosis marker, was also significantly reduced in the KB-CP20 cells as compared to the KB-3-1 cells during a period of 2 h incubation as seen in Figure 2A and B

. We observed that accumulation of Texas Red dextran-10 in the KB-3-1 cells was located mostly at the TGN (Trans-Golgi Network) region, while in KB-CP20 cells the Texas Red dextran-10 was reduced in amount and deposited at the cytoplasm near the peripheral membrane, indicating a dysfunctional uptake pathway for the marker in CP-r cells. FACS analysis on a time course of up to 7 h incubation of cells with Texas Red dextran-10 indicated a four-fold more uptake in the KB-3-1 cells (Figure 2C) than in the KB-CP20 cells (Figure 2D) during the incubation period. A relative semiquantitative measurement based on the data from the FACS analysis is shown in Figure 2E.

### Measurement of EGF binding, uptake, release and degradation

**Figure 3:**
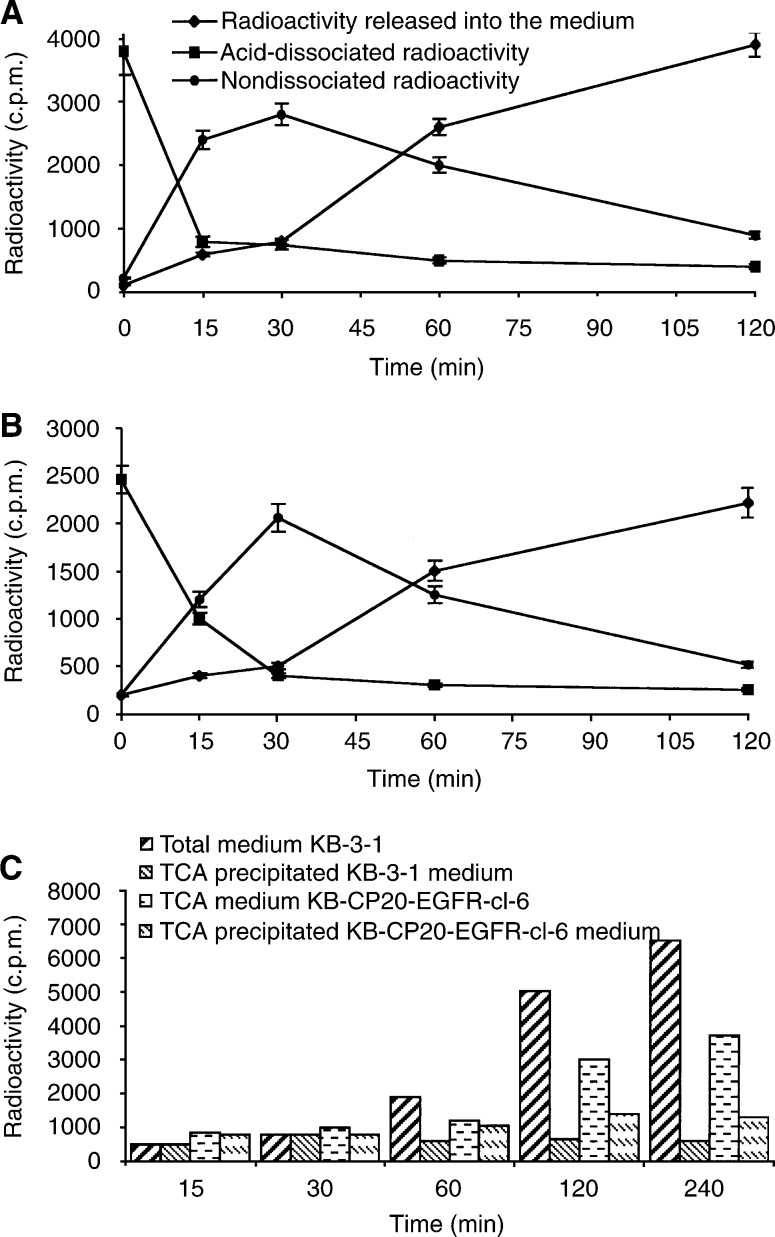
Kinetics of ^125^I-EGF uptake, degradation and release into the medium by KB-3-1 (**A**) and KB-CP20 cells (**B**). Details of the experimental procedure are given in Materials and Methods. Values are mean ±s.d. from three independent experiments. (**C**) Kinetics of TCA precipitable ^125^I-EGF release by KB-3-1 and KB-CP20 cells. Total medium at different time points of ^125^I-EGF uptake was divided into two equal parts. Total radioactivity was determined in one half and the radioactivity in the TCA-precipitated fraction of the remaining half was also determined. Values expressed here are the mean of two independent experiments.

We used ^125^I-EGF uptake to measure receptor-mediated endocytosis in KB-3-1 and KB-CP20 cells. The binding of radiolabelled EGF on the cell surface was at least three-fold less in KB-CP20 cells as compared to the KB-3-1 cells, indicating fewer functional EGF receptors on their surface. This result has been confirmed by quantitation of EGF receptors in the plasma membrane as determined by Western blot (data not shown). In order to compensate for the reduced number of EGF receptors on the surface of KB-CP20 cells, these cells were transfected with EGF receptor cDNA. A clone (KB-CP20-EGFR-cl-6) with receptor number more comparable to KB-3-1 cells was used for the following experiments. As shown in Figure 3A and B

, both KB-3-1 and KB-CP20-EGFR-cl-6 cells internalised the majority of the surface bound ligand within 30 min (nondissociated radioactivity). Similarly, the release of radioactive ligand into the culture medium after internalisation followed similar kinetics. However, after 120 min, for KBCP-20EGFR-cl-6 cells 45% of the radioactive ligand released into the medium, after internalisation, could be precipitated by TCA compared to only 12% in the case of KB 3-1 cells (Figure 3C). These results indicate that degradation of internalised radiolabelled EGF is more efficient in CP-s cells than in the resistant cells, suggesting that CP-r cells may have a defect in lysosomal acidification.

### Secretion and processing of procathepsin L

**Figure 4:**
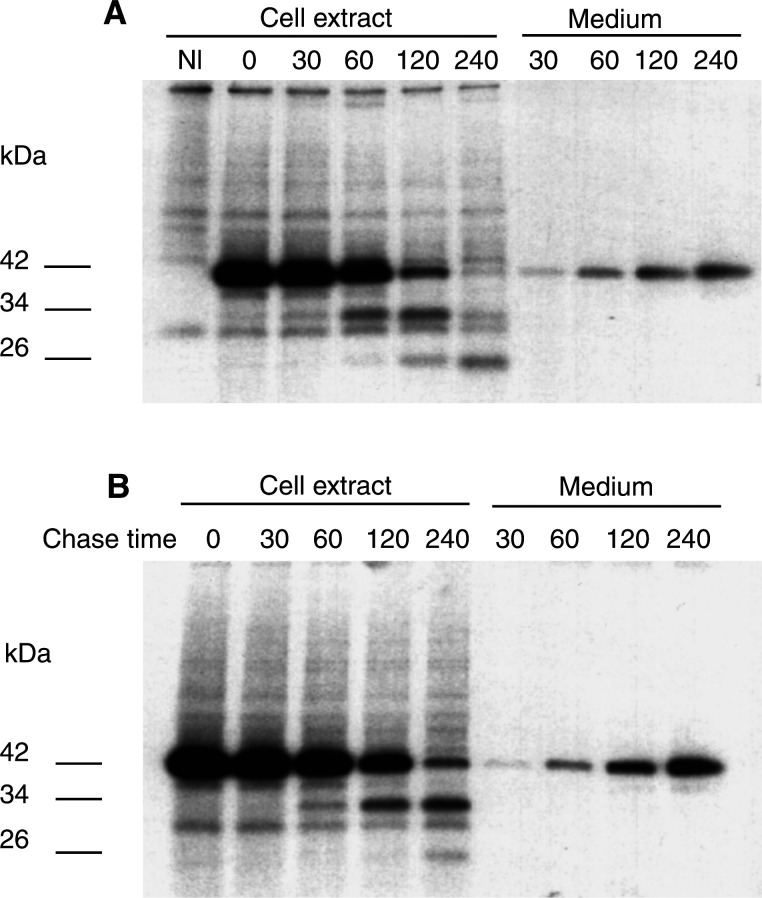
Kinetics of human procathepsin L secretion and processing in KB-3-1 (**A**) and KB-CP20 (**B**) cells. Cells were radiolabelled briefly for 30 min with Trans-^35^S-label and chased for varying time periods (0 – 240 min, as shown on the top of the gel panels A and B) in serum-free medium. At the end of each time period, medium was saved and cells were lysed after washing with ice-cold PBS. The radiolabelled human procathepsin L was immunoprecipitated using a polyclonal antibody. The immunoprecipitates were resolved on SDS – PAGE and subjected to fluorography. The values given on the left side of the panels indicate the molecular weight of different forms of cathepsin L in kDa.

Malignantly transformed cells are known to secrete a large amount of the 42 kDa procathepsin L into the culture medium (Miesenbock *et al* 1998). To understand if cellular secretion of this protease is affected by the development of the CP-r phenotype in KB-3-1 cells, we studied the kinetics of procathepsin L secretion into the culture medium by KB-3-1 and KB-CP20 cells. Our results demonstrate that both KB-3-1 and KB-CP20 cells secrete procathepsin L into the culture medium in comparable amounts (Figure 4A, B

). However, the kinetics of processing of procathepsin L is different in the two cell lines. In KB-3-1 cells, the unprocessed 34 kDa cathepsin L is detectable in the first 30 min of the chase period and the 26 kDa mature cathepsin L appears at 60 min. In these cells, the majority of the 42 kDa procathepsin L form is processed to the 26 kDa mature form of cathepsin L by 240 min, and the 34 kDa form is barely detectable at this period. In contrast, in KB-CP20 cells the 34 kDa band appears after a 60 min chase, and the 26 kDa mature cathepsin L is barely detectable even after 240 min. At this time in KB-CP20 cells, all three forms of cathepsin L (42, 34 and 26 kDa bands) are detectable, the 34 kDa form being predominant. However, at 240 min, the 26 kDa form is predominant in KB-3-1 cells and the other two forms of cathepsin L are barely detectable. These results indicate that the rate of processing of 42 kDa procathepsin L into its enzymatically active 26 kDa mature form is dramatically slowed in KB-CP20 as compared to the sensitive parental cells. However, the secretory pathway for procathepsin L appears to be intact.

### Bafilomycin A_1_ treatment and uptake of ^14^C-carboplatin and HRPO

**Figure 5:**
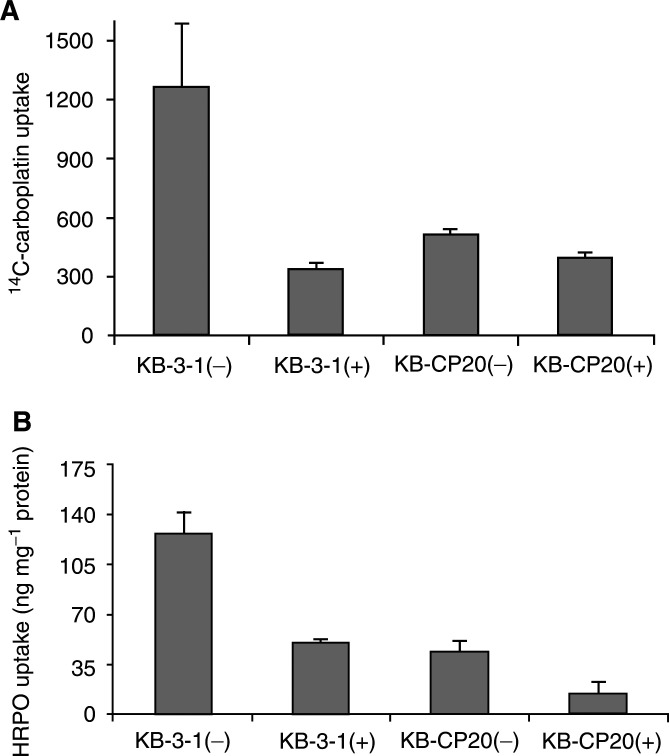
Effect of bafilomycin A_1_ treatment on the uptake of ^14^C-carboplatin (**A**) and HRPO (**B**) by CP-r and CP-s cells. Cells were washed twice with prewarmed serum-free DMEM and incubated at 37°C with the same medium in the presence (+) or absence (−) of 1 *μ*M bafilomycin A_1_. After 30 min medium was removed and fresh DMEM containing ^14^C-carboplatin (2 *μ*Ci ml^−1^) or HRPO (2 mg ml^−1^) with (+) or without (−) bafilomycin A_1_ was added. Then the incubation was continued at 37°C in a CO_2_ incubator. After 1 h for ^14^C-carboplatin uptake and 2 h for HRPO uptake, cells were processed as described in Materials and Methods. Values are mean ±s.d. from three independent experiments.

Ligands internalised by receptor-mediated endocytosis may be degraded by proteases in the lysosomal compartment. The above results suggested a possible lysosomal defect in the CP-r cells. Since acidic lysosomal pH plays a very important role in the processing of proteases and degradation of proteins in this compartment, we altered lysosomal/endosomal pH by treatment with bafilomycin A_1_ and studied its effect on the uptake of ^14^C–carboplatin by CP-r and CP-s cells. The results are given in Figure 5A

. Treatment with bafilomycin A_1_ (1 *μ*M) resulted in a four-fold decrease in the accumulation of ^14^C-carboplatin by KB-3-1 cells. This treatment resulted in little or no decrease in the accumulation of ^14^C-carboplatin in KB-CP20 cells. We observed a 2 – 2.5-fold decrease in the uptake of HRPO (Figure 5B) by bafilomycin A_1_ treatment in both CP-r and CP-s cells. These results demonstrate that blocking of lysosomal/endosomal acidification in cells has effects similar to the phenotype of CP-r on uptake of HRPO and ^14^C-carboplatin in KB-3-1 cells. Additional studies showed that bafilomycin treatment of KB-3-1 cells also blocked the degradation of ^125^I-EGF as was seen in untreated KB-CP20 cells (data not shown).

### Changes of lysosome pH in CP-r cells

**Figure 6:**
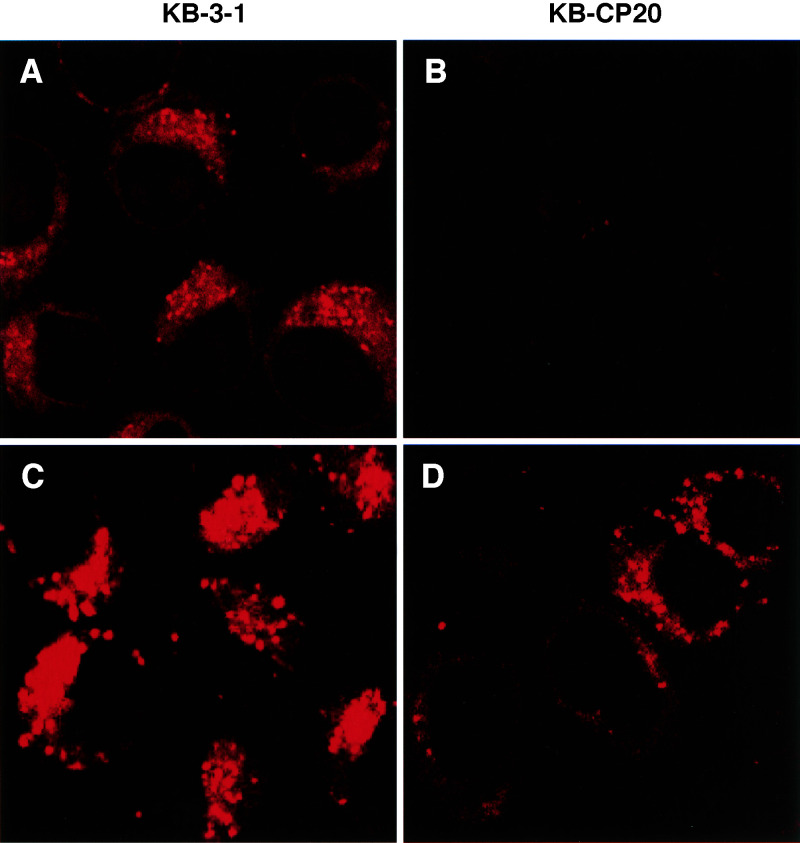
Confocal microscopic fluorescence images of lysosomal pH (**A**, **B**) by LysoSensor DND 198 and lysosomes (**C**, **D**) by LysoTracker Red DND-99 in KB-3-1 and KB-CP20 cells. (A, C): KB-3-1 cells; (B, D): KB-CP20 cells.

A pH-sensitive fluorescent dye LysoSensor DND 198 (Molecular Probes, Inc., Eugene, OR, USA) was used to determine the intracellular lysosomal pH in living cells. The intracellular fluorescence intensity was greatly decreased in the CP-r cells (Figure 6A, B

), consistent with an increase in lysosomal pH in the resistant cells. Use of LysoTracker Red DND-99, which is a lysosomal dye not sensitive to pH, showed the presence of lysosomes in the CP-r cells (Figure 6D), although perhaps somewhat decreased in number in comparison to the CP-s cells (Figure 6C). Using the cellubrevin pHluorins, it was found that the sensitive cells have an average ratio of 1.1 – 1.4 (corres-ponding to an endosomal pH of 5.5 – 6.0). The resistant cells have an average ratio of 2.0 – 2.6, corresponding to an endosomal pH of 7.2 – 7.5 (data not shown), suggesting an alkalinisation of the endosomes in the CP-r cells.

### Crossresistance of CP-r cells and *Pseudomona*s exotoxin (PE)-resistant KB cells and reduced accumulation of ^14^C-carboplatin

**Figure 7:**
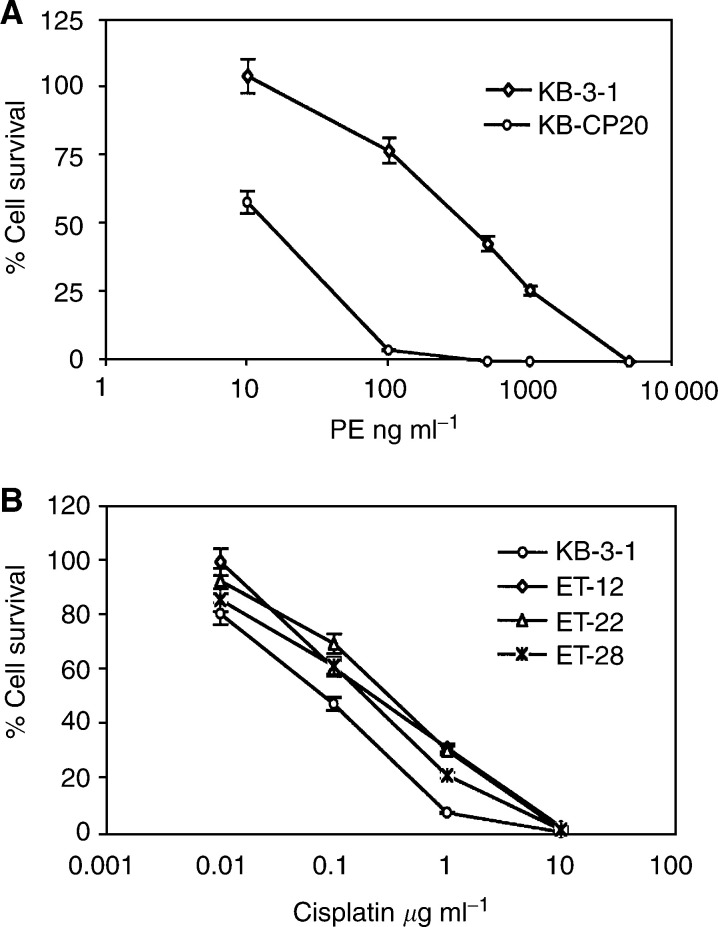
Killing curves of KB-3-1 and KB-CP20 cells with unconjugated PE (**A**), and human *Pseudomonas* exotoxin-resistant cell lines ET-12, ET-22 and ET-28 to cisplatin in comparison to their wild-type KB-3-1 cells (**B**) were determined as described in Materials and Methods.

**Figure 8:**
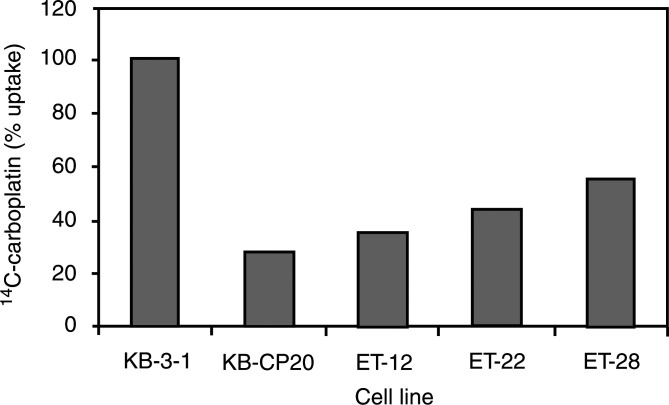
Uptake assay of ^14^C-carboplatin. Cells were incubated with ^14^C-carboplatin at 37°C for 1 h. Radioactivity was measured as described in Materials and Methods.

The killing curves shown in Figure 7A

indicate that the KB-CP20 cells were about 12-fold more resistant than the KB-3-1 cells to PE. Furthermore, cell lines previously selected for resistance to *Pseudomonas* toxin (ET cells) (Amano *et al*, 1998) were also more crossresistant to cisplatin by four-fold compared to the parental wild-type KB-3-1 cells (Figure 7B). Uptake assays of ^14^C-carboplatin in these ET cells demonstrate a significant reduction in accumulation in these ET cell lines (Figure 8

).

## DISCUSSION

Cisplatin is a potent anticancer drug, but its clinical effectiveness is undermined by the inherent and acquired resistance of tumour cells to this drug. Although the mechanism of CP-r has been postulated to be multifactorial, decreased accumulation of this drug has been consistently observed in resistant tumour cells (Naredi *et al*, 1994). Previously, we and others observed reduced uptake of cisplatin or its analogue carboplatin in CP-r cells, with no difference in the efflux of this drug between sensitive and resistant cells (Loh *et al*, 1992; Mistry *et al*, 1992; Shen *et al*, 2000). These studies indicate that defective uptake of CP was one of the mechanisms responsible for the resistance. In the present study, receptor-mediated and fluid-phase endocytosis were assessed using the well-characterised ligand EGF to understand the molecular basis of reduced CP uptake leading to CP-r. Fluid-phase endocytosis, as measured by uptake of HRP and Texas Red dextran-10, was found to be reduced in CP-r cells. While receptor-mediated endocytosis for EGF was intact, trafficking of EGF to lysosomes and/or lysosomal degradation of EGF appeared to be defective in CP-r cells.

We have previously reported a pleiotropic defect in the expression of many cell surface proteins in CP-r cells (Shen *et al*, 1998). The decreased uptake and cell surface binding of radiolabelled EGF by CP-r cells observed in the present study suggests the downregulation of this receptor on the cell surface or its decreased synthesis in these cells. Western blot analysis revealed decreased levels of the EGF receptor in CP-r cells (unpublished data), an observation consistent with our previous results (Mistry *et al*, 1992). However, expression of the EGF receptor in KB-CP20 cells at a level comparable to the KB-3-1 cells also did not result in a proportionate increase in cell surface binding of radiolabelled EGF (data not shown). These results suggest that in addition to the decreased synthesis of EGF receptor, CP-r cells may also harbour a defect in targeting it to the cell surface or in membrane protein recycling. In recent studies (Liang XJ, Shen DW, Garfield S, and Gottesman MM, submitted), we have clearly demonstrated a defect in cell surface localisation for two additional cell surface proteins.

In receptor-mediated endocytosis, ligand–receptor complexes are internalised and transported via clathrin-coated vesicles to the endosomes. Many endocytosed ligands including EGF dissociate from their receptor in the acidic environment of endosomes and are finally degraded in the lysosomal compartment (Maxfield and Yamashiro, 1991). Approximately 90% of the endocytosed ^125^I-EGF is degraded into monoiodotyrosine (which cannot be precipitated by TCA) by human fibroblasts in 0.5–2.0 h (Carpenter and Cohen, 1976). Our results (Figure 3) suggest that the degradation of endocytosed EGF is not as efficient in KB-CP20 cells as in KB-3-1 cells. Since internalised EGF is degraded in the lysosomal compartment, decreased degradation in the resistant cells could be because (i) internalised EGF is not reaching the lysosomal compartment, (ii) the lysosomal compartment in KB-CP20 cells does not contain proteases and (iii) functionally inactive proteases are present in the lysosomes.

We have previously demonstrated that KB-3-1 cells express large quantities of cathepsin L, a lysosomal cysteine protease, the majority of which is secreted into the culture medium (Chauhan *et al*, 1998). In the present study, we used cathepsin L to compare the expression of lysosomal proteases in KB-3-1 and KB-CP20 cells, and our results demonstrate that both CP-r and CP-s cells express and secrete this protease at comparable levels (unpublished data). Thus, these results rule out the possibility of proteases being absent in CP-r cells and demonstrate that there is no defect in the secretory mechanism of these cells. Human cathepsin L is synthesised as a 42 kDa preproenzyme and autoprocessed into a 34 kDa proenzyme and enzymatically active 26 kDa forms in the lysosomes (Salminen and Gottesman, 1990; Chauhan *et al*, 1998). Lysomotropic agents like NH_4_Cl and chloroquine inhibit the processing of this enzyme to smaller forms, indicating the requirement of acidic pH for this purpose (Chauhan S and Gottesman MM, unpublished results). In the present study, we observed delayed processing of cathepsin L in the lysosomes of CP-r cells (Figure 5), consistent with the reduced degradation of ^125^I-EGF.

Bafilomycin A_1_, a macrolide antibiotic that has been demonstrated to inhibit vacuolar type H^+^-ATPase, inhibits endosomal and lysosomal acidification and blocks lysosomal degradation of EGF in A431 cells (human epidermoid carcinoma cells) (Yoshimori *et al*, 1991; Melikova *et al*, 2001), blocked degradation of ^125^I-EGF in KB-3-1 cells. Therefore, since acid lysosomal pH is essential for proper modification, processing and trafficking of proteins, we wished to determine whether the inefficient acidification of endosomal/lysosomal compartment(s) was responsible for the inefficient degradation of ^125^I-EGF in KB-CP20 cells. Using a pH-sensitive fluorescence-labelled probe (LysoSensor DND-189, Molecular Probes, Eugene, OR, USA), a reduction in lysosomal pH and intensity of lysosomal staining was detected in KB-CP-r cells as compared to the KB-3-1 cells. Thus, a defect in lysosomal acidification may play a role in the improper processing of cathepsin L as well as inefficient degradation of internalised ^125^I-EGF in CP-r cells. Additional data also show that the early endosome compartment in CP-r cells is more basic than in the CP-s cells, while the Golgi complex appears to have a pH similar to the CP-s cells. In a recent publication, the intracellular pH of CP-r cells was also found to be significantly higher than that of the sensitive parental cells (Murakami *et al*, 2001).

Low endosomal pH has been demonstrated to be essential for *Pseudomonas* exotoxin cytotoxicity in mammalian cells (Pastan *et al*, 1992). Inhibitors of endosomal acidification protect cells from diphtheria toxin and *Pseudomonas* exotoxin cytotoxicity (Olsnes and Sandvig, 1988; Pastan *et al*, 1992). Lyall *et al* (1987) isolated human clones of KB-3-1 cells resistant to EGF–*Pseudomonas* exotoxin conjugates. In the present study, these cells were found to be crossresistant to CP (Figure 7A). Similarly, CP-r cells were found to be crossresistant to *Pseudomonas* exotoxin (Figure 7B) and to show reduced accumulation of ^14^C-carboplatin (Figure 8). These results suggest that the endosomal/lysosomal acidification defect may be responsible for some of the observed CP-r in KB-CP20 cells.

Conclusions of this study are in agreement with a previous report, which demonstrated increased sensitivity of tumour cells to cisplatin at lower cellular pH (Laurencott *et al*, 1995). The involvement of defective vacuolar acidification in CP-r was further confirmed by the four-fold reduction in carboplatin uptake by KB-3-1 cells after bafilomycin A**_1_** treatment. In addition to reduced carboplatin uptake, treatment of KB-3-1 cells with bafilomycin A_1_ blocked ^125^I–EGF degradation and HRPO uptake (Figure 5). Our results clearly demonstrate that CP-r cells, which exhibit reduced uptake of ^14^C carboplatin and HRPO, harbour an endosomal acidification defect (Figures 1, 2, 3, 4, 5 and 6). Blocking endosomal/lysosomal acidification by bafilomycin A_1_ in KB-3-1 cells mimics the CP-r phenotype in terms of ^14^C-carboplatin and HRPO uptake. Therefore, we conclude that defective endosomal/lysosomal acidification may, at least, partly be responsible for CP-r due to its reduced uptake, resulting in less drug reaching the cytotoxic targets.

## References

[bib1] Amano F, Gottesman MM, Pastan I (1998) Epidermal growth factor-dependent growth of human KB cells in a defined medium and altered growth factor requirements of KB mutants resistant to EGF – *Pseudomonas* exotoxin conjugates. J Cell Physol 26: 502–508 10.1002/jcp.1041350319 3294236

[bib2] Carpenter G, Cohen S (1976) ^125^I-labelled human epidermal growth factor: binding internalization, degradation in human fibroblasts. J Cell Biol 71: 159–171 977646 10.1083/jcb.71.1.159 PMC2109737

[bib3] Chauhan SS, Ray D, Kane SE, Willingham MC, Gottesman MM (1998) Involvement of carboxy-terminal amino acids in the secretion of human lysosomal protease cathepsin L. Biochemistry 37: 8584–8594 9622510 10.1021/bi972251z

[bib4] Chu G (1994) Cellular responses to cisplatin. The role of DNA binding proteins and DNA repair. J Biol Chem 269: 787–790 8288625

[bib5] Das M, Chauhan SS, Misra VS, Sanger JM, Sanger JW, Roy-Chaudhary S (1989) Aberrant post endocytotic fate of a 34-kDa molecular mass growth factor from human trophoblasts. Cancer Res 4: 2761–2765 2713859

[bib6] DeFeudis P, D'Incalci M, Broggini M (1996) Block of bcr – abl expression and induction of apoptosis by *cis*-platinum in human chronic myeloid leukemia cell line. Apoptosis 1: 161–166

[bib7] Fanidi A, Harrington EA, Evan GI (1993) Cooperative interaction between c-myc and bcl-2 proto-oncogenes. Nature 359: 554–556 10.1038/359554a0 1406976

[bib8] Fink D, Aebi S, Howell SB (1998) The role of DNA mismatch repair in drug resistance. Clin Cancer Res 4: 1–6 9516945

[bib9] Godwin AK, Meister A, Anderson ME (1992) High resistance to cisplatin in human ovarian cancer lines is associated with marked increase of glutathione synthesis. Proc Natl Acad Sci USA 89: 3070–3074 1348364 10.1073/pnas.89.7.3070 PMC48805

[bib10] Gosland S, Lum B, Schimmelpfennig J, Baker J, Doukas M (1996) Insights into mechanisms of cisplatin resistance and potential for its clinical reversal. Pharmacotherapy 16: 16–39 8700790

[bib11] Hettinga JVE, Konings AW, Kampinga HH (1997) Reduction of cellular cisplatin resistance by hyperthermia – a review. Int J Hypertherm 13: 439–457 10.3109/02656739709023545 9354931

[bib12] Husain A, He G, Venkatraman ES, Spriggs DR (1998) BRCA 1 up-regulation is associated with repair mediated resistance to *cis*-diaminedichloroplatinum(II). Cancer Res 58: 1120–1123 9515792

[bib13] Ishida S, Lee J, Thiele DJ, Herskowitz I (2002) Uptake of the anticancer drug cisplatin mediated by the copper transporter Ctr 1 in yeast and mammals. Proc Natl Acad Sci USA 99: 14298–14302 12370430 10.1073/pnas.162491399 PMC137878

[bib14] Johnson SW, Shen DW, Pastan I, Gottesman MM, Hamilton TC (1996) Cross resistance, cisplatin accumulation, and platinum – DNA adduct formation and removal in cisplatin-sensitive and -resistant human hepatoma cell lines. Exp Cell Res 226: 133–139 8660948 10.1006/excr.1996.0211

[bib15] Katano K, Kondo A, Safaei R, Holzer A, Samimi G, Mishima M, Kuo YM, Rochdi M, Howell SB (2002) Acquisition of resistance to cisplatin is accompanied by changes in the cellular pharmacology of copper. Cancer Res 62: 6559–6565 12438251

[bib16] Kelly SL, Basu A, Ticher BA, Hacker MP, Hammer DH, Lazo JS (1988) Overexpression of metallothionein confers resistance to anticancer drugs. Science 241: 1813–1815 3175622 10.1126/science.3175622

[bib17] Kondo Y, Kuo SM, Watkin SC, Lazo JS (1995) Metallothionein localization and cisplatin resistance in human hormone-independent prostatic tumor cell lines. Cancer Res 55: 474–477 7834610

[bib18] Lai SL, Hwang J, Perng RP, Whang-Peng J (1995) Modulation of cisplatin resistance in acquired-resistant non small cell lung cancer cells. Oncology Res 7: 31–38 7549042

[bib19] Laurencott CM, Andrews PA, Kennedy KA (1995) Inhibitors of intracellular pH regulation induce cisplatin resistance in EMT 6 mouse mammary tumor cells. Oncol Res 7: 363–369 8747599

[bib20] Loh SY, Mistry P, Kelland LR, Abel G, Harrap KR (1992) Reduced drug accumulation as a major mechanism of acquired resistance to cisplatin in a human ovarian carcinoma cell line: circumvention studies using novel platinum (II) and (IV) ammine/amine complexes. Br J Cancer 66: 1109–1115 1457352 10.1038/bjc.1992.419 PMC1978040

[bib21] Lowe SW, Ruley HE, Jacks T, Housman DE (1993) p53 dependent apoptosis modulates the cytotoxicity of anticancer agents. Cell 74: 975–967 10.1016/0092-8674(93)90719-7 8402885

[bib22] Lyall RM, Hwang J, Cardarelli C, FitzGerald D, Akiyama S, Gottesman MM, Pastan I (1987) Isolation of human KB cell lines resistant to epidermal growth factor –*Pseudomonas* exotoxin conjugates. Cancer Res 47: 2961–2966 3494506

[bib23] Maxfield FR, Yamashiro DJ (1991) Acidification of organelles and the intracellular sorting of proteins during endocytosis. In Intracellular Trafficking of Proteins, Steer CJ, Hanover JA (eds) pp 157–182. Cambridge, UK: Cambridge University Press

[bib24] Melikova MS, Blagoveshchenskaya AD, Nikolsky NN, Kornilova ES (2001) Influence of vacuolar proton pump inhibitor bafilomycin A1 on intracellular processing of receptor-mediated and fluid phase endocytosis markers. Abstract in 41st American Society for Cell Biology Annual Meeting, Washington DC, 8-12 December pp 344a–345a

[bib25] Miesenbock G, De Angelis DA, Rothman JE (1998) Visualizing secretion and synaptic transmission with pH-sensitive green fluorescent proteins. Nature 394: 192–195 9671304 10.1038/28190

[bib26] Minn AJ, Rudin CM, Boise LH, Thompson CB (1995) Expression of bcl-xL can confer a multidrug resistance phenotype. Blood 86: 1903–1910 7655019

[bib27] Mistry P, Kelland LR, Loh SY, Abel G, Murrer BA, Harrap KR (1992) Comparison of cellular accumulation and cytotoxicity of cisplatin with that of tetraplatin and amminedibutyratodichloro-(cyclohexylamine)-platinum(IV) (JM1221) in human ovarian carcinoma cell lines. Cancer Res 52: 6188–6193 1423261

[bib28] Moorehead RA, Singh G (2000) Influence of proto-oncogene c-fos on cisplatin sensitivity. Biochem Pharmacol 59: 337–345 10644041 10.1016/s0006-2952(99)00333-0

[bib29] Moscow JA, Cowan KH (1998) Multidrug resistance. J Natl Cancer Inst 80: 14–20 10.1093/jnci/80.1.14 2892943

[bib30] Murakami T, Shibuya I, Ise T, Chen ZS, Akiyama S, Nakagawa M, Izumi H, Nakamura T, Matsuo K, Yamada Y, Kohno K (2001) Elevated expression of vacuolar protein pump genes and cellular pH in cisplatin resistance. Int J Cancer 93: 869–874 11519050 10.1002/ijc.1418

[bib31] Naredi P, Heath DD, Enns RE, Howell SB (1994) Cross-resistance between cisplatin and antimony in a human ovarian carcinoma cell line. Cancer Res 52: 6464–6468 7987844

[bib32] Niedner H, Christen R, Lin X, Howell SB (2001) Identification of genes that mediate sensitivity to cisplatin. Mol Pharmacol 60: 1153–1160 11723219

[bib33] Olsnes S, Sandvig K (1988) How protein toxins enter and kill cells. In Immunotoxins, Frankel AE (ed) pp. 39–73. Dordrecht, the Netherlands: Kluwer Academic Publishers 10.1007/978-1-4613-1083-9_4 2908634

[bib34] Ozols RF, Williams SD (1989) Testicular cancer. Curr Prob Cancer 13: 287–335 10.1016/0147-0272(89)90020-2 2551577

[bib35] Pastan I, Chaudhary V, Fitgerald DJ (1992) Recombinant toxins as novel therapeutic agents. Annu Rev Biochem 61: 331–354 1497314 10.1146/annurev.bi.61.070192.001555

[bib36] Perez RP (1998) Cellular and molecular determinants of cisplatin resistance. Eur J Cancer 34: 1535–1542 9893624 10.1016/s0959-8049(98)00227-5

[bib37] Salminen A, Gottesman MM (1990) Inhibitor studies indicate that active cathepsin L is probably essential to its own processing in cultured fibroblasts. Biochem J 272: 39–44 2264836 10.1042/bj2720039 PMC1149653

[bib38] Shen DW, Akiyama S-I, Schoenlein P, Pastan I, Gottesman MM (1995) Characterization of high-level cisplatin-resistant cell lines established from a human hepatoma cell line and human KB adenocarcinoma cells: cross-resistance and protein changes. Br J Cancer 71: 676–683 7710928 10.1038/bjc.1995.134 PMC2033730

[bib39] Shen DW, Goldenberg S, Pastan I, Gottesman MM (2000) Decreased accumulation of ^14^C-carboplatin in human cisplatin-resistant cells results from reduced energy-dependent uptake. J Cell Physiology 183: 108–116 10.1002/(SICI)1097-4652(200004)183:1<108::AID-JCP13>3.0.CO;2-4 10699972

[bib40] Shen DW, Pastan I, Gottesman MM (1998) Cross-resistance to methotrexate and metals in human cisplatin-resistant cell lines results from a pleiotropic defect in accumulation of these compounds associated with reduced plasma membrane binding proteins. Cancer Res 58: 268–275 9443404

[bib41] Simonian PL, Grillot DAM, Nunez G (1997) Bcl-2 and BcL-XL can differentially block chemotherapy-induced cell death. Blood 90: 1208–1216 9242554

[bib42] West MA, Bretscher MS, Watts C (1989) Distinct endocytotic pathways in epidermal growth factor-stimulated human carcinoma A431 cells. J Cell Biol 109: 2731–2739 2556406 10.1083/jcb.109.6.2731 PMC2115909

[bib43] Yoshimori T, Yamamoto A, Moriyama Y, Futai M, Tashiro Y (1991) Bafilomycin A, a specific inhibitor of vacuolar-type H+-ATPase, inhibits acidification and protein degradation in lysosomes of cultured cells. J Biol Chem 266: 17707–17712 1832676

[bib44] Zaman GJ, Lankelma J, van Tellingen O, Beijnen J, Dekker H, Planlusma C, Oude Elferink RP, Baas F, Borst P (1995) Role of glutathione in the export of compounds from cells by the multidrug-resistance-associated protein. Proc Natl Acad Sci USA 92: 7690–7694 7644478 10.1073/pnas.92.17.7690 PMC41211

[bib45] Zhen M, Link Jr CJ, O'Connor PM, Reed E, Parker P, Howell SB, Bohr VA (1992) Increased gene-specific repair of cisplatin interstrand cross-links in cisplatin-resistant human ovarian cancer cell lines. Mol Cell Biol 12: 3689–3698 1380646 10.1128/mcb.12.9.3689-3698.1992 PMC360224

